# *bla*_KPC-24_-Harboring Aeromonas veronii from the Hospital Sewage Samples in China

**DOI:** 10.1128/spectrum.00555-22

**Published:** 2022-05-12

**Authors:** Chengcheng Yang, Min Guo, Haizhen Yang, Yicheng Wen, Zhichen Zhu, Tao Wang, Jie Zhu, Liang Chen, Hong Du

**Affiliations:** a Department of Clinical Laboratory, The Second Affiliated Hospital of Soochow University, Suzhou, Jiangsu, People’s Republic of China; b Health Management Center, The Second Affiliated Hospital of Soochow University, Suzhou, Jiangsu, People’s Republic of China; c Hackensack Meridian Health Center for Discovery and Innovation, Nutley, New Jersey, USA; d Department of Medical Sciences, Hackensack Meridian School of Medicine, Nutley, New Jersey, USA; Universidad de Buenos Aires, Facultad de Farmacia y Bioquímica

**Keywords:** *Aeromonas veronii*, IncP-6 plasmid, KPC-24, carbapenemase variants, hospital sewage

## Abstract

KPC-24, different from KPC-2 by a single amino acid alteration at codon 6 (R6P), was initially discovered in Klebsiella pneumoniae in Chile. Here, we reported KPC-24-producing Aeromonas veronii isolates from hospital sewage in China. The *bla*_KPC-24_ was cloned and the MICs were tested against β-lactams antimicrobial agents. KPC-24 exhibited a β-lactam susceptibility profile similar to that of KPC-2. Whole-genome sequencing and analysis revealed that *bla*_KPC-24_ was located within a Tn*6296*-related region on an IncP-6 plasmid.

**IMPORTANCE** Our study described a variant of K. pneumoniae carbapenemase (KPC), KPC-24, from two A. veronii strains isolated from hospital sewage, in which antibiotics, biocides, pharmaceuticals, and heavy metals may supply an appropriate condition for the evolution of carbapenemases. Some variants exhibited stronger hydrolysis activity to antibiotics and gave rise to a major public health concern. More seriously, *Aeromonas* species are prevalent in aquatic environments and, thus, may act as a suitable vector for antibiotics-resistance genes and foster the transmission of resistance. We should attach importance to surveying the evolution and transmission of antibiotics-resistance genes.

## OBSERVATION

Due to the continual release of antibiotic-resistant bacteria and antibiotic residues into the environment, hospital sewage water may act as a potential reservoir and environmental supplier of antibiotic resistance, raising significant public health concern (1). Carbapenems are considered a last-line defense of complicated infections caused by multidrug-resistant bacteria. Carbapenem resistance is mainly due to the production of different carbapenemases, which can hydrolyze a wide spectrum of β-lactam antibiotics, including penicillins, cephalosporins, and carbapenems. Since Klebsiella pneumoniae carbapenemase (KPC), a class A serine β-lactamase, was initially identified in the United States in 2001 (2), it has emerged as the predominant carbapenemase worldwide, ranging from sporadic to endemic because of its robust ability of hydrolyzing β-lactam antibiotics and a broad range of bacteria hosts.

To date, over 100 variants of KPC have been identified (http://bldb.eu/). Among them, *bla*_KPC-24_ was first identified in K. pneumoniae strains isolated from clinical samples in Chile (3). Nucleotide alignment of different *bla*_KPC_ variants showed that *bla*_KPC-24_ differs from *bla*_KPC-2_ in a single mutation (C17G), resulting in an amino acid substitution at codon 6 (R6P). In the original study, although KPC-24 had been assigned, its resistance spectrum has not been explored yet. Here, we isolated two *bla*_KPC-24_-harboring Aeromonas veronii strains and examined the antimicrobial susceptibility profile of KPC-24 through gene cloning and susceptibility testing. Whole-genome sequencing was then used to assess the *bla*_KPC-24_ surrounding genetic environment and plasmid structure.

Two *A. veronii* strains, HD6448 and HD6451, were isolated from the main sewer outlet with different depths of 0.5 to 1 m below the surface at a tertiary hospital in eastern China in July 2019. Bacterial species identification was carried out using matrix-assisted laser desorption/ionization time of flight mass spectrometry (MALDI-TOF-MS). PCR and Sanger sequencing showed that the two strains carried the carbapenem-resistant gene *bla*_KPC-24_.

In order to determine the susceptibility profile of KPC-24, we cloned the *bla*_KPC-2_ and *bla*_KPC-24_ genes along with the same native promoter sequences of *bla*_KPC-2_ into the pET28a vector. The promoter and *bla*_KPC_ regions were cloned in the opposite orientation from the T7 promoter in pET28a to avoid leaky effect of T7 RNA polymerase-mediated transcription. The recombinant plasmids pET28a-KPC-24 and pET28a-KPC-2 were transformed into Escherichia. coli DH5α via electroporation and then selected on Luria broth agar medium supplemented with kanamycin (50 μg/mL). Successful transfers of *bla*_KPC-24_ and *bla*_KPC-2_ were confirmed by PCR and Sanger sequencing. The empty vector pET28a was transformed into DH5α and used as the control.

The susceptibility to antimicrobial agents was examined by the standard broth microdilution method according to the 2020 Clinical and Laboratory Standards Institute (CLSI) guidelines (4), with E. coli ATCC 25922 as the quality control strain (QC). As shown in Table 1, there is no difference between DH5α/pET28a-KPC-2 and DH5α/pET28a-KPC-24 MICs of meropenem, imipenem, ertapenem, ampicillin, aztreonam, and ceftazidime-avibactam. However, DH5α/pET28a-KPC-24 exhibited lower MIC of ceftazidime (2-fold) than DH5α/pET28a-KPC-2.

**TABLE 1:** Antimicrobial drug susceptibility profile

Drug	MIC (mg/L) of strain				
	HD6448	HD6451	DH5α		
	(KPC-24)	(KPC-24)	(pET28a-KPC-2)	(pET28a-KPC-24)	(pET28a)
Ampicillin	>512	>512	>64	>64	<4
Aztreonam	512	512	64	64	<2
Meropenem	16	16	1	1	<0.125
Ertapenem	256	256	8	8	<0.25
Imipenem	8	8	2	2	<0.25
Ceftazidime	128	128	16	8	<1
Ceftazidime-avibactam	<2/4	<2/4	<2/4	<2/4	<2/4
Tetracycline	16	16			
Tigecycline	<0.125	<0.125			
Kanamycin	256	256			
Gentamicin	8	8			
Chloramphenicol	<1	<1			

Amino acid substitutions in KPC enzymes may lead to varied hydrolysis spectrum of antibiotics. Several mutations have been shown to increase ceftazidime resistance. For example, KPC-4, KPC-9, KPC-14, KPC-49, KPC-41, KPC-35, and KPC-28 possess decreased catalytic efficiency against carbapenems but increased ceftazidime hydrolysis (5–11). Moreover, KPC-15 expression manifested higher resistance to β-lactam antibiotics than that of KPC-2 (12).

β-lactamases are synthesized as immature precursors in the cytoplasm. The amino-terminal sequence acts as a signal peptide and is recognized by cellular sorting and translocation machinery, which leads the protein to its final destination. Signal peptides are frequently removed by specialized signal peptidases after protein delivery to the correct subcellular compartment (13). It has been speculated that the first 24 amino acids of KPC-2, which were cleaved from the mature protein, could account for the signal peptide (14).

In this study, DH5α/pET28a-KPC-24 slightly decreased its potency against ceftazidime. However, KPC-24 differs from KPC-2 in the sixth amino acid, located at the signal peptide region. Therefore, the slight MIC alteration in DH5α/pET28a-KPC-24 cannot be explained by the hydrolysis activity of the KPC enzyme.

To assess the genetic environment of *bla*_KPC-24_, the two strains were subjected to Illumina sequencing. The Omega Bio-Tek bacterial DNA kit (Doraville, GA, USA) was used to extract bacterial genomic DNA according to the manufacturer’s instructions. Nanopore sequencing was further conducted in strain HD6448 (Bioproject no. PRJNA780685), generating completely closed chromosome and plasmid sequences.

Open reading frames (ORFs) and pseudogenes were predicted using RAST 2.0 (https://rast.nmpdr.org/) combined with BLASTP/BLASTN (https://blast.ncbi.nlm.nih.gov/Blast.cgi) searches against the UniProtKB/Swiss-Prot database (https://web.expasy.org/docs/swiss-prot_guideline.html) and the RefSeq database (https://www.ncbi.nlm.nih.gov/refseq/). Annotation of resistance genes, mobile elements, and other features was carried out using the online databases CARD (https://card.mcmaster.ca/), ResFinder 4.1 (https://cge.food.dtu.dk/services/ResFinder/), ISfinder (https://www-is.biotoul.fr/), Tn Number Registry (https://www.ucl.ac.uk/eastman/tn-number-registry), and oriTfinder (https://tool-mml.sjtu.edu.cn/oriTfinder/). Gene organization diagram was drawn in Inkscape v1.0 (https://inkscape.org/en/).

HD6448 and HD6451 carried the same resistance genes, encoding resistance to aminoglycosides [*aadA1*, *aadA16*, *aac (6′)-Ib3*, *aac(6′)-Ib-cr*], tetracyclines [*tet*(E)], phenicols [*catB3*], disinfectants [*qacE*], β-lactams [*bla*_TEM_, *ampS*, *bla*_CEPH-A3_, *bla*_KPC-24_], macrolides [*mph*(E), *msr*(E), *mph*(A)], and quinolones [*qnrS2*]. Among them, *bla*_CEPH-A3_ is a class B metallo-beta-lactamase gene, frequently found in *Aeromonas* species (15). As shown in Table 1, HD6448 and HD6451 exhibited similar MICs of gentamicin, kanamycin, chloramphenicol, ampicillin, aztreonam, ceftazidime, ceftazidime-avibactam, and tigecycline. The core single nucleotide polymorphism (SNP) analysis of both strains based on their draft genome sequences showed that their core genomes differed by only a few SNPs (*n* = 15) and further suggested that both strains belonged to a single clone.

**FIG 1:**
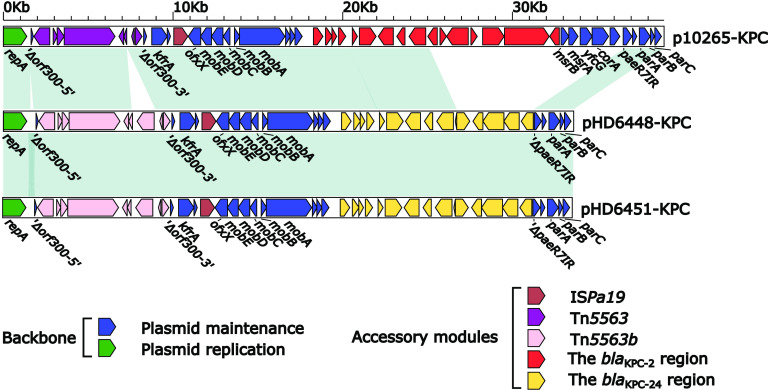
Linear comparison of IncP-6 plasmids pHD6448-KPC, pHD6451-KPC, and p10265-KPC. Genes are denoted by arrows. Genes, mobile genetic elements, and other features are colored based on function classification. Shaded regions denote homology of two plasmids (light blue: ≥99% nucleotide identity).

**FIG 2:**
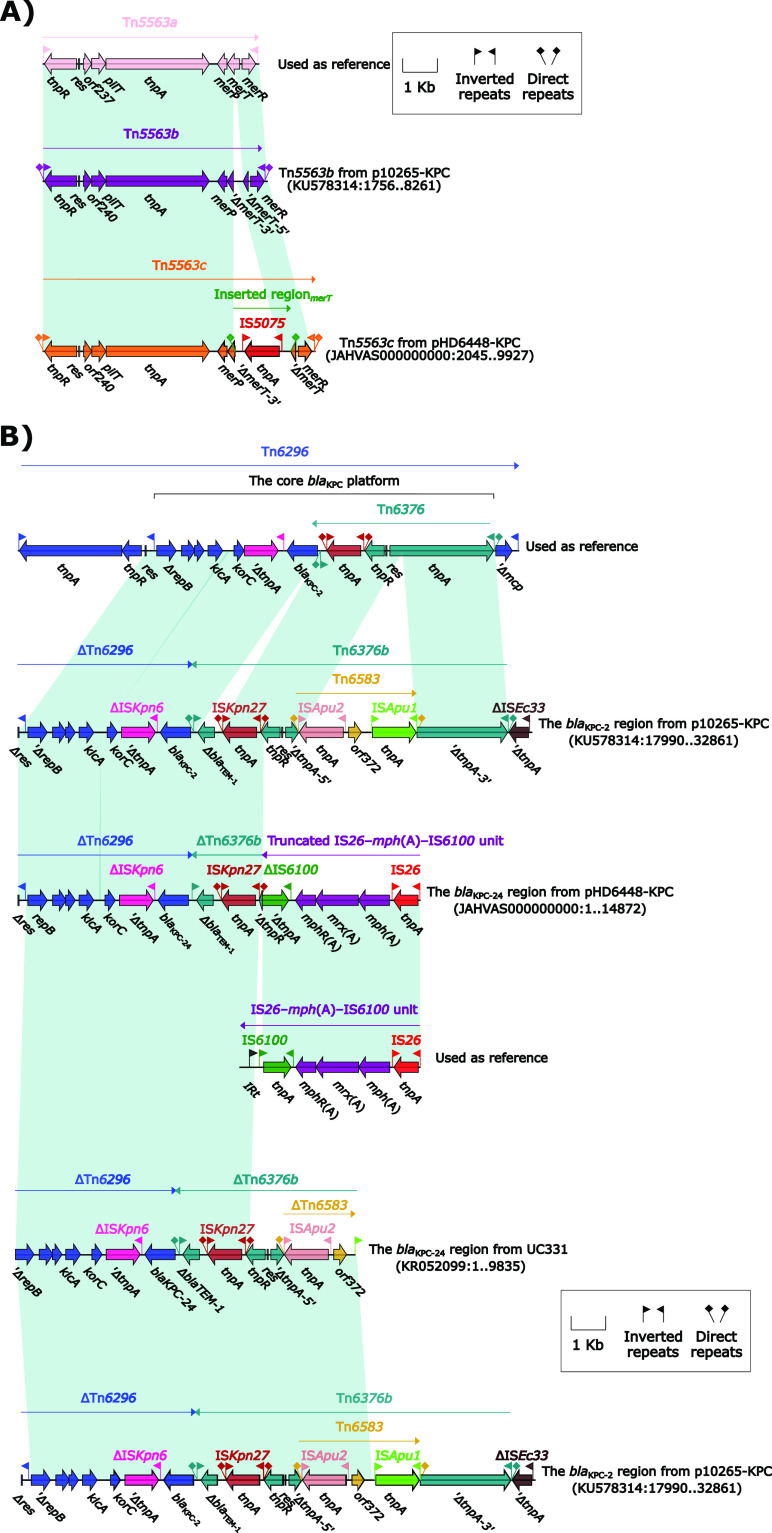
(A) Organization of Tn*5563c* from pHD6448-KPC and comparison to related genetic elements. (B) Linear comparison of the *bla*_KPC_ region. Genes are denoted by arrows. Genes, mobile genetic elements, and other features are colored based on their functional classification. Shading denotes regions of homology (light blue: ≥99% nucleotide identity). Numbers in brackets indicate nucleotide positions within the sequences. The accession number of Tn*6296* (24) used as reference is FJ628167.

The *bla*_KPC-24_ was carried by a 33.61-kb IncP-6 plasmid in HD6448, which was assigned the name pHD6448-KPC. Further plasmid assembly was obtained mapping contigs of HD6451 on pHD6448-KPC, checking overlapping paired ends, and confirming the assembly by the PCR-based gap closure method, which indicates that pHD6451-KPC was almost identical to pHD6448-KPC except for a 34-bp deletion in the backbone of the plasmid (Fig. 1). The modular structure of pHD6448-KPC consisted of the backbone and three accessory modules (Tn*5563c*, IS*Pa19*, and the *bla*_KPC-24_ region). BLASTN analysis showed that pHD6448-KPC and pHD6451-KPC are closely related to IncP-6 plasmid, p10265-KPC (accession number KU578314) (16), collected from a patient with pneumonia in a Chinese hospital. However, it is vital to notice that pHD6448-KPC carried *bla*_KPC-24_ but p10265-KPC carried *bla*_KPC-2_. The backbone of plasmid pHD6448-KPC shared 99.99% nucleotide identity to p10265-KPC with 76% coverage. Compared with the backbone of p10265-KPC, pHD6448-KPC lost a 3.8-kb sequence including *msrB*, *msrA*, *yscG*, *corA*, and part of *paeR7IR*. Additionally, the exogenous inserted region was different (Fig. 1). The transposon Tn*5563b* in p10265-KPC differed from Tn*5563a* by the insertion of an unknown region and caused a truncation of *merT-3′*. The inserted region, which was flanked by 9-bp direct repeats (DRs), was inserted to *merT* of Tn*5563c* from pHD6448-KPC in comparison with Tn*5563a*. Moreover, there was a similarity in the insertion regions inserted in *merT* of Tn*5563b* and Tn*5563c* to a certain extent (Fig. 2A). Additionally, the *bla*_KPC-2_ region from p10265-KPC was composed of a truncated Tn*6296* (carrying the *bla*_KPC-2_) and a truncated Tn*6376b*. The *bla*_KPC-24_ region from pHD6448-KPC was composed of a truncated Tn*6296* (carrying the *bla*_KPC-24_), a truncated Tn*6376b*, and a truncated IS*26–mph*(A)–IS*6100* unit. In order to compare the structure of the first reported *bla*_KPC-24_ of the Klebsiella pneumoniae strain from Chile, named UC331, with HD6448, we downloaded a partial sequence of UC331 from NCBI. The *bla*_KPC-24_ region from UC331 was almost identical to the *bla*_KPC-2_ region from p10265-KPC (Fig. 2B).

Horizontal transfer of antibiotic resistance gene (ARG) *bla*_KPC-24_ was examined by bacterial conjugation experiment using rifampin-resistant E. coli E600 as the recipient strain. We failed to obtain the transconjugants in conjugation experiments after three repetitions. Further inspection showed that pHD6448-KPC does not contain the *tra* module, which appears to be consistent with the conjugation results and the descriptions of other IncP-6 plasmids as reported previously (16, 17).

Broad-host-range IncP-6 plasmids were naturally identified in Pseudomonas aeruginosa in 1975, associated with the increased prevalence of KPC in various species (18). *Aeromonas* species are opportunistic pathogens; however, they are prevalent in aquatic environments (19), which may serve as reservoirs for the further dissemination of ARGs in the environment. Even though *bla*_KPC-24_-harboring plasmid pHD6448-KPC failed to conjugate in the conjugation experiments, the possibility of horizontal transfer cannot be ruled out. *bla*_KPC_ was usually associated with mobile genetic elements (MGEs) that are involved in horizontal gene transfer (HGT) among Gram-negative bacteria. One possibility is that the acquisition of *bla*_KPC-24_ in IncP-6 plasmid is due to MGE-mediated transposition or recombination from coexisting plasmids or chromosomes. Additionally, a nonconjugative plasmid may be mobilizable through its Mob apparatus with the assistance of a conjugative helper plasmid. Since the complete sequence of the Chile *bla*_KPC-24_ plasmid is not available, we cannot evaluate the evolutionary relationship between the two *bla*_KPC-24_ plasmids. However, pHD6448-KPC was highly similar to other *bla*_KPC-2_-harboring IncP-6 plasmids (e.g., p10265-KPC) found in China. Given this, one more possible explanation is that *bla*_KPC-24_ evolved from the mutation of *bla*_KPC-2_ on an IncP-6 plasmid.

High density of bacteria, biofilms, and stress caused by pollutant compounds such as antibiotics, biocides, pharmaceuticals, and heavy metals in sewage may increase the probability of ARGs evolution, leading to variant subtype exhibiting analogical or altered hydrolysis spectrum (20). In fact, some variants already showed increased catalytic efficiency, causing public health concern with predictions of catastrophic developments in the coming years (12). The widespread detection of *bla*_KPC_ and the related variants in aquatic environments is an alarming environmental issue with potentially serious public health implications. On the contrary, ARGs in the environment may circulate from wastewater to human, demanding efficacious measures to prevent their spread (21). Sewage supplied special and ideal settings for the mutation and mobilization of ARGs (22). On one hand, a diverse mixture of antibiotics and other pollutants exerted significant selection pressures on bacteria, and on the other hand, high levels of ARGs as well as elements for HGT, including plasmids, insertion sequences, transposons, integrons, genomic islands, integrating conjugative elements, and bacteriophages, promoted the spread of ARGs and the exchange of MGEs (23). Thus, a close surveillance policy should be enacted to control and limit the evolution and transmission of ARGs.

### Data availability.

The sequence data of HD6448 and HD6451 are available from the NCBI with accession numbers CP087266 to CP087271 and JAHVAR000000000, respectively.
